# Combined treatment of sorafenib and doxorubicin-loaded microbubble-albumin nanoparticle complex for hepatocellular carcinoma: A feasibility study

**DOI:** 10.1371/journal.pone.0243815

**Published:** 2020-12-11

**Authors:** Seunghyun Lee, Jung Hoon Kim, Hyungwon Moon, Hak Jong Lee, Joon Koo Han

**Affiliations:** 1 Department of Radiology, Seoul National University Hospital, Jongno-gu, Seoul, Republic of Korea; 2 Department of Radiology, Seoul National University College of Medicine, Jongno-gu, Seoul, Republic of Korea; 3 Institute of Radiation Medicine, Seoul National University Medical Research Center, Jongno-gu, Seoul, Republic of Korea; 4 IMGT Co., Ltd., Bundang-gu, Seongnam, Republic of Korea; 5 Department of Radiology, Seoul National University Bundang Hospital, Bundang-gu, Seongnam, Republic of Korea; Texas A&M University, UNITED STATES

## Abstract

**Purpose:**

To assess the feasibility of the combined sorafenib (SOR) and doxorubicin-loaded microbubble-albumin nanoparticle complex (DOX-MAC) treatment effect in an orthotopic rat model of hepatocellular carcinoma (HCC).

**Materials and methods:**

Sixty-two rats with N1-S1 hepatoma were divided into four groups according to the treatment methods, i.e. G1 (SOR and DOX-MAC; n = 12), G2 (SOR; n = 15), G3 (DOX-MAC; n = 12), G4 (DOX; n = 11), and G5 (normal saline; n = 12). We performed the theragnostic, contrast-enhanced ultrasound examination and treatment at the baseline, one-week, and two-weeks. Tumor volume and perfusion parameters were compared at each time point and the differences between all of the groups over time were analyzed using repeated measures ANOVA. We also analyzed the apoptotic index and microvessel density (MVD) per each tumor specimen in all of the groups.

**Results:**

The tumors increased from the beginning in all of the groups to the final follow-up, whereas the tumor growth in the G1 group and the G2 group was inhibited during the treatment period compared to the baseline tumor volume (*P* = 0.016 and *P* = 0.031). The G1 group resulted in tumor growth inhibition compared to the control group (*P* = 0.008). The G1 group showed that the peak enhancement and wash-in area under the curve were lower than that of the G4 group (*P* = 0.010 and 0.022). However, there was no difference in perfusion parameters in the other treated group compared to control group. The MVD of the G1 group tumor was lower than that of the G4 group (*P* = .016).

**Conclusion:**

Our results suggest that the combination therapy of SOR and DOX-MAC can cause inhibition of tumor growth after treatment and that this therapy can be adequately monitored using the theragnostic DOX-MAC agent.

## Introduction

Systemic chemotherapy has been proposed as a treatment option for patients with advanced hepatocellular carcinoma (HCC) [1]. Doxorubicin (DOX) is a commonly used cytotoxic agent for the treatment of HCC [2]. Sorafenib (SOR) is also one of the molecular-targeted agents with antiangiogenic effects for the treatment of advanced HCC [1]. The combination of SOR and DOX is feasible and effective in enhancing the effects of SOR, but the previous reports have reported shorter overall patient survival and higher toxicity than single-agent therapy [2,3]. Recently, the targeted drug delivery strategy has been suggested to locally deliver DOX while using drug carriers such as nanoliposome and stimuli-responsive material such as microbubble (MB) [4]. For the delivery of a chemotherapeutic agent, doxorubicin-loaded, microbubble-albumin nanoparticle complex (DOX-MAC) can be burst by applying high ultrasonic acoustic pressure which leads to the release of DOX from DOX-MAC. Therefore, DOX-MAC has a potential for in vivo uses for contrast-enhanced ultrasound (CEUS) imaging with MB, which was one of a component, and local delivery of DOX to minimize the toxicity of the chemotherapeutic agent [5]. Therefore, our study investigated the efficacy of combination therapy with SOR and DOX-MAC and the feasibility of DOX-MAC as drug delivery in an orthotopic rat HCC model. We also evaluated the feasibility of CEUS for the monitoring tool using DOX-MAC or MAC as theragnostic agents.

## Materials and methods

This study was approved by our Institutional Animal Care and Use Committee (IACUC; No. 17-0113-S1A0(1)) and was performed in accordance with the Guide for our IACUC and the National Institute of Health Guide for the Care and Use of Laboratory Animals.

### Preparation of DOX-loaded Microbubble-albumin nanoparticle complex (MAC)

The microbubble-albumin nanoparticle complex (MAC) consisted of albumin nanoparticles on the MB with fabrication by the following procedures. First, we synthesized albumin nanoparticle using the desolvation technique [6], which was dissolved human serum albumin (Sigma-Aldrich, St. Louis, MO, USA) at a concentration of 50 mg/mL in distilled water. We adjusted this solution for pH 8.5 by 0.1M of NaOH, dropped 8 mL of ethanol at 1 mL/min, and added 50 μL of glutaraldehyde (8%) for cross-linking. We purified the albumin nanoparticles, removed the large size of aggregates, and collected the supernatant for albumin nanoparticles.

And then we synthesized DOX (Sigma-Aldrich, St. Louis, MO, USA) loaded albumin nanoparticles (DOX-NPs) by mixing albumin and DOX with the 1:10 of the mass ratio for 2-hour. We stirred albumin and DOX with 600 rpm, added ethanol as a solvent to the albumin and DOX solution, and added 50μL-8% glutaraldehyde for cross-linking. We centrifuged the albumin and DOX mixture with 15,000 rpm for 15 min at 4°C to remove unbound DOX and albumin at three times. We quantified unbound DOX using HPLC [7], and measured the size distribution and zeta potential of DOX-NPs using dynamic light scattering.

We synthesized MBs using phospholipid and sulfur hexafluoride gas (SF_6_). We dissolved 1,2-distearoyl-sn-glycero-3-phosphocholine (DSPC, NOF, Japan) and 1,2-distearoyl-sn-glycero-3-phosphoethanolamine-N-[succinyl(polyethylene glycol)-2000] (DSPE-PEG2k-NHS, NOF, Japan) in chloroform at the molar ratio of 9:1. We added 1mL of the phospholipid mixture to the 2 mL-vial with filling the headspace of the vial by SF_6_ gas. The phospholipid solution was transformed to MBs by the agitation using VialMIX^TM^ (Lantheus Medical Imaging, N. Billerica, MA, USA) for 45s [8].

**Fig 1:**
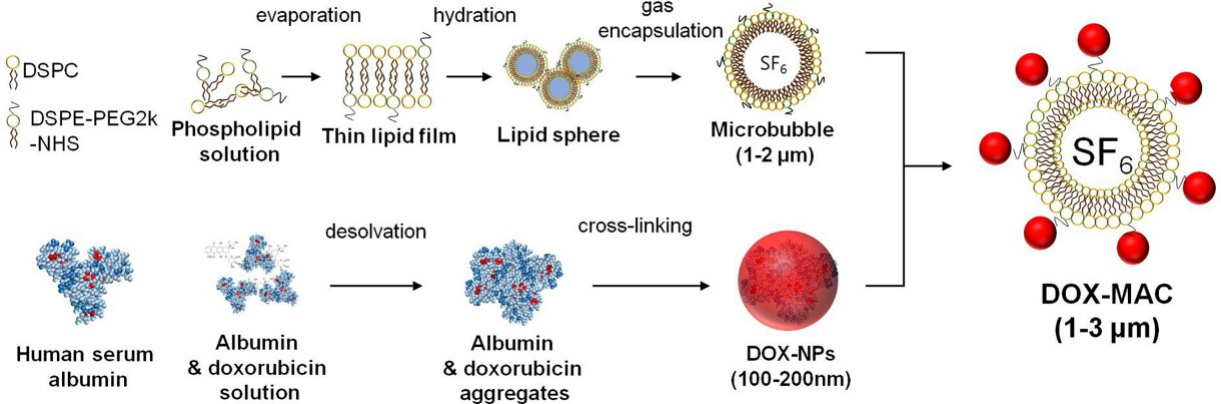
Synthesis of microbubble (MB), doxorubicin binding human serum albumin nanoparticle (DOX-NPs) and DOX-NPs conjugated microbubble complex (DOX-MAC).

After MB fabrication, we added 200μL-DOX-NPs or albumin-NPs to the MB solution for the manufacture of DOX-MAC or MAC. We induced the conjugation of DOX-NPs or albumin-NPs to the surface of the microbubble via an amide bond between the NHS of the MB and NH_2_ of each of the nanoparticles. We performed the conjugation by gently shaking the vial for 0.5–1 hour. We measured the size distribution of MAC and DOX-MAC by dynamic light scattering (Malvern Zetasizer NanoZS90, UK). The **Fig 1** shows the process of the DOX-MAC synthesis.

### Animal models

We obtained the N1-S1 (CRL-1604; ATCC, Manassas, VA, USA) tumor cell line which was prepared using the RPMI-1640 (WelGENE, Daegu, Korea) with 10% fetal bovine serum and a 1% penicillin/streptomycin mixture (Gibco, Grand Island, NY, USA). We assessed cell viability with Trypan blue staining to confirm cell viability of > 90% before tumor implantation.

We used male Sprague-Dawley rats weighing approximately 400 g in this study. Rats were housed in the standard animal care facility cage during all experiments, which kept on a natural dark and light (12:12 h) cycle, in a temperature and humidity-controlled room and had free access to food and rodent food pellets. Under intraperitoneal general anesthesia using a mixture of 5 mg/kg of zolazepam (Zoletil; Virbac, Carros, France) and xylazine hydrochloride (Rompun 2%; Bayer Korea, Seoul, Korea), we performed mini-laparotomy to expose the left lateral lobe of the liver. According to the established protocols for the N1-S1 tumor model, we injected the N1-S1 cell lines (5 × 10^6^ cells prepared in 50 μL of the medium) at the exposed left lateral lobe of the liver [9,10]. We performed a two-layer abdominal incision closure in order that the rats would survive. We injected Cyclosporine A (20 mg/kg/day; Chong Kun Dang Pharmaceutical Corp., Seoul, Korea) one day before tumor implantation to four days postoperatively to prevent spontaneous regression of N1-S1 cells [11]. After the surgery, the animals were transferred to their home cage, which kept on a natural dark and light cycle in a temperature and humidity-controlled room. The rats were allowed access to food and pellets after recovery from anesthesia with strict observation for the first 2 hours after surgery. Rats were received daily postoperative care with regular food intake amount and body weight measured during the follow-up period after surgery. The rats had be planned euthanasia when a decrease in food intake for three days or weight loss of 20%. From the seventh day after each cell line injection, tumor induction and growth were monitored every third day using GE LOGIQ E9 ultrasound equipment (GE Healthcare, Wauwatosa, WI, USA). After confirming tumor growth of up to 10 mm on ultrasound imaging, the rats underwent subsequent treatment studies.

### Experimental protocol

**Fig 2:**
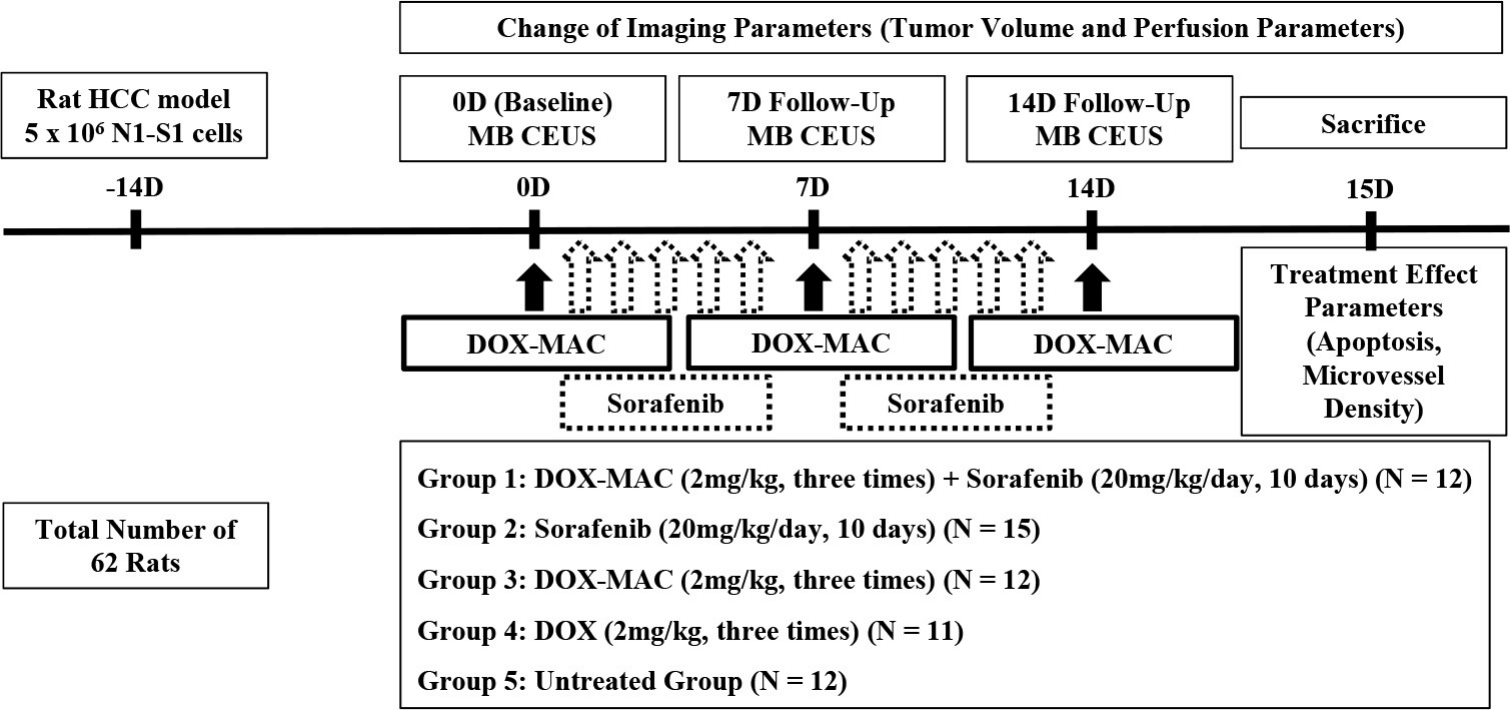
Study design. Experimental design for the animal study, showing the timeline of each group for N1-S1 cell inoculation, doxorubicin-loaded microbubble-albumin nanoparticle complex (DOX-MAC) with/without sorafenib and doxorubicin (DOX) therapy, microbubble (MB) CEUS imaging, and animal sacrifice for tumor harvest as described in the “Materials and Methods” section.

**Fig 2** summarizes the experiment schedule. We distributed randomly 62 rats with N1-S1 hepatoma to the SOR and DOX-MAC combination-treated group (G1; n = 12), the SOR ttreated group (G2; n = 15), the DOX-MAC treated group (G3; n = 12), the DOX-treated group (G4; n = 11, one rat was excluded due to accidental image loss), and the untreated control group (G5; n = 12). We performed CEUS using DOX-MAC or MAC alone for simultaneous treatment and monitoring the therapeutic effect at the baseline, one-week, and two-week follow-up (____with 18 years of clinical experience) using the GE LOGIQ E9 Ultrasound System (GE Healthcare, Wauwatosa, WI, USA).

First, we measured the tumor volume at the baseline, one week and two weeks after the treatment start. We measured the tumor size to determine its longitudinal (a) and short (b) diameters, and estimated the tumor volume (mm^3^) according to the modified ellipsoidal formula (volume = 1/2ab^2^). Second, we performed a CEUS examination with the following parameters: a transducer frequency of 9-MHz; a frame rate of 13 Hz; a dynamic range of 60; a mechanical index of 0.14; a gain of 24; a depth of 2.0 cm; and scan time of 90 seconds. The ultrasound contrast agent (DOX-MAC or MAC) was intravenously injected at a dose of 0.2 mL and was followed with flushing using 0.5 mL saline, using a power injector with the rate of 1 mL/min. We obtained the dynamic CEUS images for 90 seconds for analysis of the perfusion parameters.

The dose of DOX in the DOX-MAC was an equivalent dosage to that of 2 mg/kg in the G1 and G3 group. Many studies decided the dosage of 5 mg/kg as a control group to compare the treatment effect, but we lowered to 2 mg/kg, the effective dose of SOR and DOX compared with the previous preclinical studies for lengthening the treatment period [12,13]. We administered SOR (Nexavar Bayer Health Care, Leverkusen, Germany) orally at a dosage of 20 mg/kg/d for two weeks in the G1 and the G2 group. The dose of SOR was selected based on previous studies and adjusted to the optimal survival dose, 20mg/kg, for maintaining each subject for two weeks [14,15]. For oral administration, SOR was dissolved in Cremophor EL/ethanol (50:50; Sigma Cremophor EL, 95% ethanol), as described in a previous study [15]. We injected DOX (2 mg/kg) via the tail vein after the CEUS examination in the G4 group. We also injected the normal saline after the CEUS examination in the G5 group without any treatment agent.

### Dynamic contrast-enhanced ultrasound perfusion analysis

We analyzed the time-intensity curve (TIC) using quantitative perfusion analysis software (VueBox, Bracco, Milano, Italy) with the quality of fit > 75 % on the dynamic CEUS images. We drew a region of interest (ROI) manually along the tumor without a necrotic portion five times in each tumor and then averaged values discarding the maximum and minimum values on the dynamic CEUS images. We also drew additional ROIs for healthy liver tissue and then obtained the mean value in the same way.

The following perfusion parameters were obtained: peak enhancement (PE); wash-in area under the curve (WiAUC); rising time (RT); mean transit time local (mTTl); time to peak (TTP); wash-in rate (WiR); and wash-in perfusion index (WiPI). We normalized the dynamic CEUS perfusion values of tumors by dividing by those of normal liver parenchyma. For all of the ROIs parameters from the quantification tool box were collected as follows: normalized peak enhancement (nPE) [a.u]; normalized wash-in area under the curve (nWiAUC) [a.u]; and normalized wash-out AUC (nWoAUC) [a.u].

### Histologic analysis

Rats were sacrificed with a lethal dose of sodium pentobarbital (100 mg/kg body weight, intraperitoneal). Just after sacrifice, the tumors of each rat were collected for histologic analysis. The specimens were fixed in 10% neutral buffered formalin, placed in paraffin, and cut into 4-μm sections. Each section was stained with the terminal, deoxynucleotidyl, transferase-mediated, dUTP nick end-labeling (TUNEL) assay in order to quantify the apoptotic cells using an apoptosis detection kit (Millipore, Bedford, MA, USA). Immunohistochemical staining for endothelial antigens was performed using the CD31 antibody (ab28364, 1:50 Abcam, Cambridge, UK) and the polymer anti-rabbit antibody (K4003, Dako).

We calculated the apoptotic index (%) as the ratio of TUNEL positive cells to the total number of cells for each of the five, randomized areas in the high-power field (×400) [15]. We also selected the five, randomized hot spots, i.e. areas of higher vascular density compared with the rest of the tissue per individual section at the low-power field (×40) and then counted the CD31-stained vessels (microvessel density, MVD) at high magnification (×200, 0.739 mm^2^) according to the method of Weidner et al. [16]. Two researchers (____ and ____) identified the necrotic area, TUNNEL positive, and CD31 positive cells with consensus, and calculated these values in randomly selected images using Image J software (National Institutes of Health, Bethesda, MD, USA).

### Statistical analysis

All statistical analyses were performed using SPSS version 21.0 (SPSS, Chicago, IL, USA). The tumor volume and perfusion parameters of each group were analyzed using one-way analysis of variance (ANOVA) with Bonferroni’s correction for multiple comparisons at each time point. Repeated measures ANOVA was performed to determine what change in tumor volume and perfusion parameters would be significant between all of the groups over time. The histological features of each group, including the apoptotic index and MVD, were compared using ANOVA with Bonferroni’s correction for multiple comparisons at each time point.

## Results

### Serial changes of tumor volume

**Fig 3:**
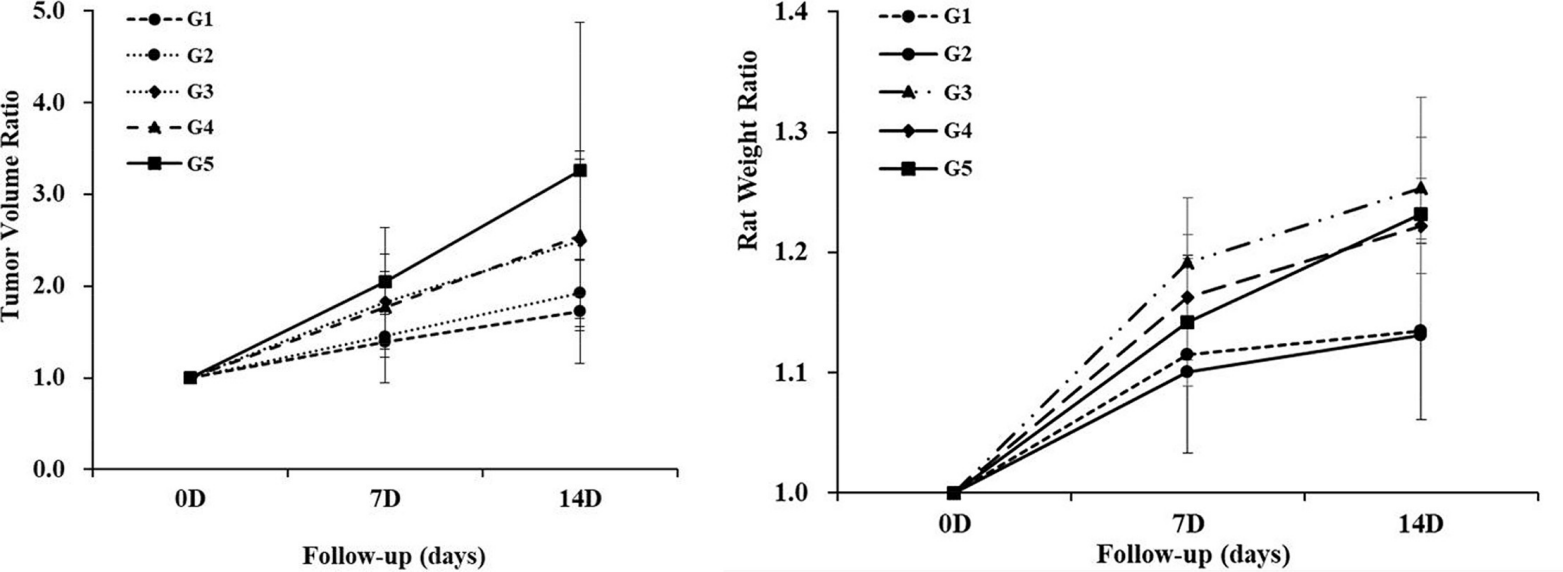
Serial changes of the relative tumor growth and rat weight. (A) The tumor volume on a specific day divided by the baseline tumor volume showed the most delayed tumor growth at two weeks in the G1 group (*P* = 0.008). (B) The rat weight on a specific day divided by the baseline weight showed the slow increasing tendency of the rat two weeks in the G1 group. However, there was no significant difference between the control group and the G1 group (*P* > 0.05).

**Table 1** summarizes the serial changes of tumor volume. We observed tumor volume increase with time in all of the groups. The maximum tumor volume was 39.0 mm^3^ in the control group at the two-week follow-up. There was no significant difference in the baseline tumor volume in any of the groups (*P* = 0.568). However, the tumor volume of the G1 group showed the most inhibition of tumor growth compared to that of the control group at the two-week interval (16.8 ± 3.8 mm^3^ vs. 27.8 ± 9.9 mm^3^, *P* = 0.008). Other treatment groups showed no difference in the tumor volume compared to the control group at a two-week follow-up. **Fig 3A** illustrates the tumor growth rate in each group. The G1 group showed 1.39 and 1.72 times at the one-week and two-week follow-up compared to the baseline tumor volume. The G2 group showed 1.46 and 1.93 times, the G3 group showed 1.83 and 2.49 times, and the G4 group showed 1.77 and 2.54 times at the one-week and two-week follow-up. The control group showed a markedly increase in tumor growth of 2.05 and 3.26 times compared to the baseline tumor volume. The tumors increased from the beginning in all of the groups to the final follow-up, whereas the tumor growth in the G1 group and the G2 group was inhibited during the treatment period compared to the baseline tumor volume (*P* = 0.016 and *P* = 0.031).

**Table 1:** Serial changes of the tumor volume.

Variable	G1	G2	G3	G4	G5	*P*-value*
	(N = 12)	(N = 15)	(N = 12)	(N = 11)	(N = 12)	
**Baseline**	10.3 ± 3.0	10.4 ± 1.4	8.5 ± 2.4	10.3 ± 2.9	9.5 ± 4.5	0.568
**1-week**	13.8 ± 3.9	15.0 ± 2.6	14.8 ± 3.7	17.6 ± 3.4	18.2 ± 6.0	0.178
**2-week**	16.8 ± 3.8*	19.7 ± 3.2	20.5 ± 7.9	24.5 ± 4.1	27.8 ± 9.9*	**0.009**

Values are mean ± SD (mm^3^). G1, doxorubicin-microbubble and sorafanib combination treated group; G2, sorafenib treated group; G3, doxorubicin-microbubble treated group; G4, doxorubicin treated group; G5, untreated control group.

*One-way analysis of variance (ANOVA).

### Serial changes of rat weight

**Table 2** summarizes the serial changes of rat weight. There was no significant difference in the baseline rat weight in any of the groups (*P* = 0.322). There was no difference in the baseline, 1 week and 2 weeks of the rat weight in each group. There was also no difference between the groups in terms of the time in any of the groups (all *P* > 0.05). **Fig 3B** illustrates the rat weight rate in each group. The G1 group showed 1.12 and 1.13 times at the one-week and two-week follow-up compared to the baseline rat weight. The G2 group showed 1.10 and 1.13 times, the G3 group showed 1.19 and 1.25 times, and the G4 group showed 1.16 and 1.22 times at the one-week and two-week follow-up.

**Table 2:** Serial changes of the rat weight.

Variable	G1	G2	G3	G4	G5	*P*-value*
	(N = 12)	(N = 15)	(N = 12)	(N = 11)	(N = 12)	
**Baseline**	286.3 ± 27.8	285.3 ± 11.9	275.9 ± 26.7	267.6 ± 20.3	279.2 ± 27.0	0.322
**1-week**	317.9 ± 21.3	314.0 ± 18.1	327.8 ± 21.5	310.7 ± 21.9	318.0 ± 21.7	0.620
**2-week**	323.5 ± 20.4	322.4 ± 17.3	345.3 ± 28.6	326.5 ± 21.6	342.3 ± 22.5	0.234

Values are mean ± SD (g). G1, doxorubicin-microbubble and sorafanib combination treated group; G2, sorafenib treated group; G3, doxorubicin-microbubble treated group; G4, doxorubicin treated group; G5, untreated control group.

*One-way analysis of variance (ANOVA).

### Serial changes of the dynamic ceus perfusion parameters

**Fig 4:**
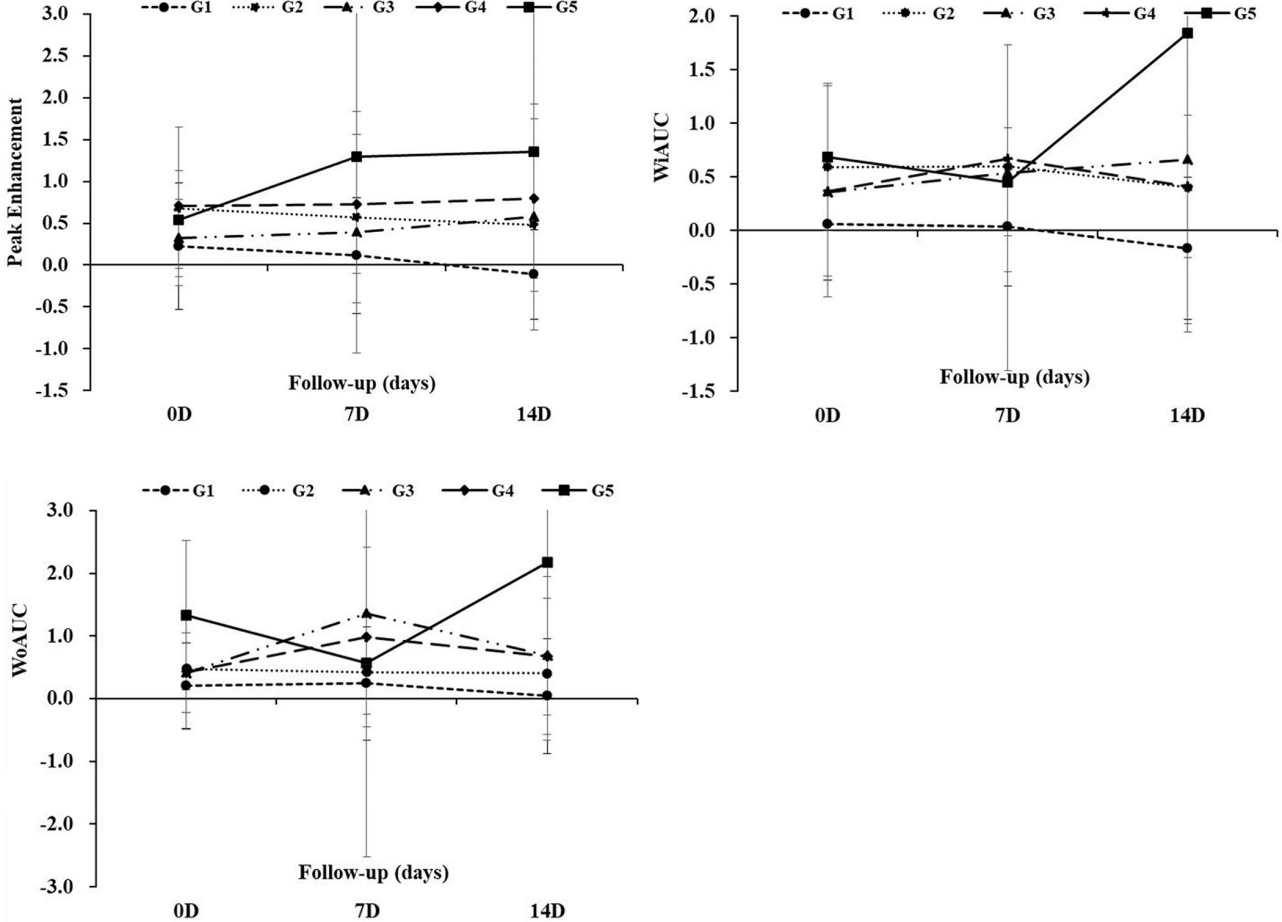
Serial changes of the perfusion parameters. (A) The normalized peak enhancement (nPE) difference between the G1 group and the G5 group increased from baseline to two weeks (*P* = 0.010) (B) The normalized wash-in area under the curve (nWiAUC) difference between the G1 group and the G5 group increased from baseline to two weeks (*P* = 0.022). (C) There was no difference in the normalized wash-out area under the curve (nWoAUC) value between the G1 and the G5 group (G1 vs. G5, *P* = 0.05).

**Table 3** summarizes the serial changes of the normalized CEUS perfusion parameters. Among the 62 rats, the dynamic CEUS images in 39 rats (62.9%) were available for the perfusion parameter analysis. The G1 group showed a decrease of nPE and nWiAUC value at a two-week compared to the baseline value. There was also a decrease of nWoAUC value in the G1 group at two-week compared to the one-week value. However, there was no change of the nPE, nWiAUC, and nWoAUC values in the other treated groups. The control group showed an increase of the nPE value at a two-week compared to the baseline value. There was also a decrease of the nWiAUC and nWoAUC values at the two-week compared to those of the baseline values, although there were exceptional values at the one-week follow-up in the control group. The difference in the nPE value between the G1 group and the control group became increased from baseline to the two-week follow-up **(Fig 4A)**. There was also an increase in the difference of the nWiAUC and nWoAUC values between the G1 group and the control group from the baseline to the two-week follow-up **(Fig 4B and 4C)**. There was a significant difference in the nPE value and the nWiAUC value between the G1 and the control group using the repeated measures ANOVA (*P* = 0.010 and *P* = 0.022).

**Table 3:** Serial changes of the normalized contrast-enhanced perfusion parameters.

Variable	G1	G2	G3	G4	G5	*P*-value*
	(N = 10)	(N = 10)	(N = 6)	(N = 7)	(N = 6)	
**nPE [a.u]**						
**Baseline**	0.23 ± 0.75	0.67 ± 0.11	0.32 ± 0.46	0.70 ± 0.95	0.54 ± 0.58	0.439
**1-week**	0.12 ± 0.69	0.57 ± 0.15	0.40 ± 1.44	0.73 ± 0.31	1.30 ± 1.75	0.261
**2-week**	**-0.11 ± 0.53**^**†**^	0.48 ± 0.14	0.58 ± 1.35	0.79 ± 0.31	**1.35 ± 1.76**^**†**^	0.072
**nWiAUC [a.u]**						
**Baseline**	0.06 ± 0.52	0.59 ± 0.12	0.36 ± 0.78	0.37 ± 0.98	0.69 ± 0.69	0.310
**1-week**	0.04 ± 0.56	0.60 ± 0.45	0.54 ± 1.84	0.67 ± 1.06	0.45 ± 0.50	0.626
**2-week**	**-0.17 ± 0.66**^**††**^	0.40 ± 0.11	0.66 ± 1.60	0.41 ± 0.67	**1.84 ± 2.71**^**††**^	0.074
**nWoAUC [a.u]**						
**Baseline**	0.21 ± 0.68	0.48 ± 0.27	0.41 ± 0.64	0.42 ± 0.91	1.33 ± 1.19	0.082
**1-week**	0.24 ± 0.90	0.42 ± 0.17	1.36 ± 3.88	0.99 ± 1.43	0.56 ± 0.81	0.723
**2-week**	**0.04 ± 0.92**^**†††**^	0.40 ± 0.20	0.69 ± 1.25	0.67 ± 0.93	**2.17 ± 2.83**^**†††**^	0.059

Values are mean ± SD. G1, combined sorafenib and doxorubicin-microbubble group; G2, sorafenib treated group; G3, doxorubicin-microbubble group; G4, doxorubicin treated group; G5, untreated control group; nPE, normalized peak enhancement; nWiAUC, normalized wash-in area under the curve; nWoAUC, normalized wash-out area under the curve.

*One-way analysis of variance (ANOVA).

^†^Repeated measures ANOVA with Bonferroni’s correction, G1 vs. G4, *P* = 0.010

^††^G1 vs. G4, *P* = 0.022

^**†††**^G1 vs. G4, *P* = 0.050.

### Histologic parameters

**Fig 5:**
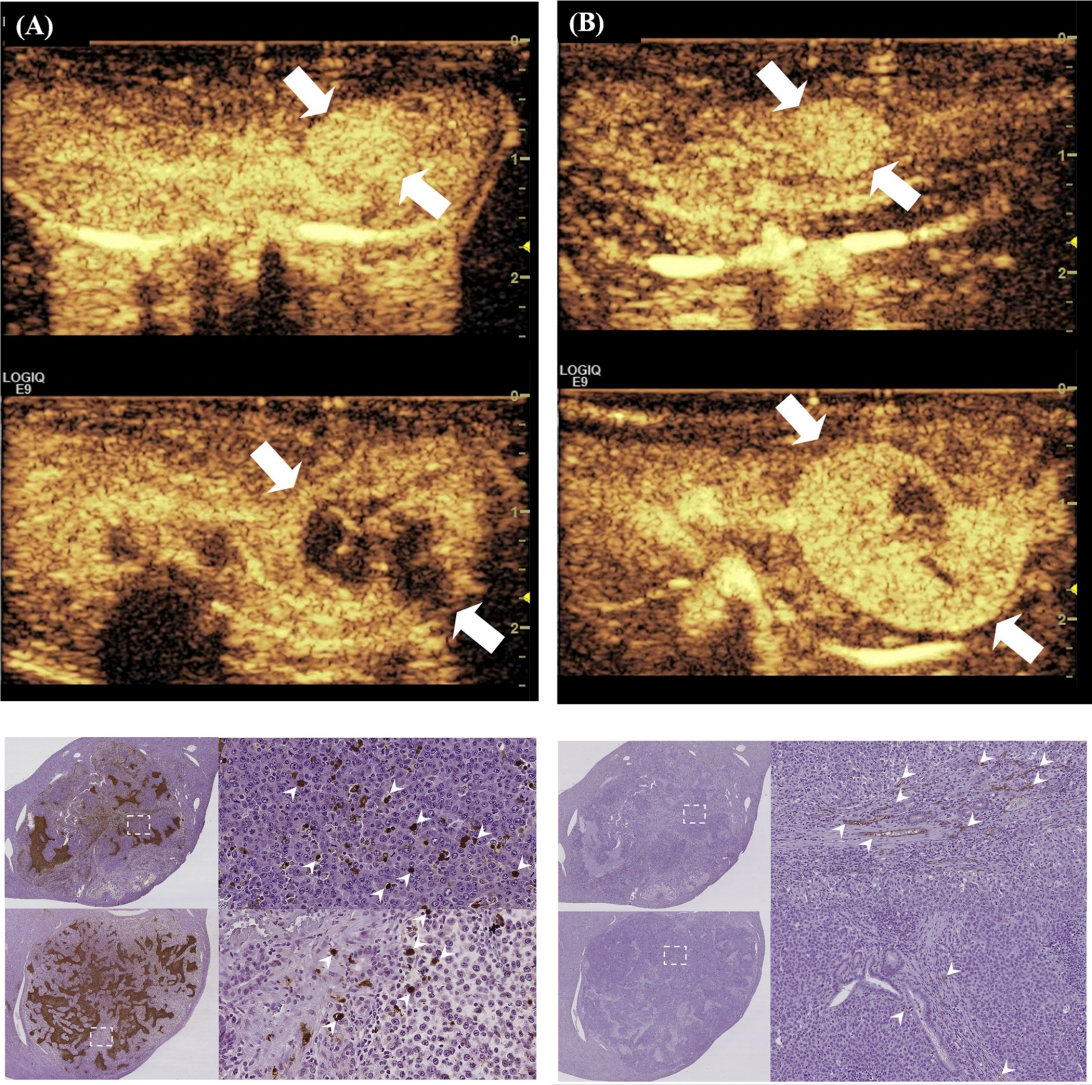
Representative cases of the G1 and G4 groups. (A) The contrast-enhanced ultrasound showed the hyper-vascular tumor in the left lobe of the liver (upper, arrows). The mass showed a decrease of vascularity after combination treatment of sorafenib (SOR) and doxorubicin-loaded microbubble-albumin nanoparticle complex (DOX-MAC) (lower, arrows). (B) One of the G5 group showed the small hyper-vascular tumor in the left lobe of the liver (upper, arrows). The mass showed an increase of tumor volume with hyper-vascularity without any treatment (lower, arrows). (C) The number of brown-colored stained apoptotic cells on TUNEL staining (×400) was a subtle increase in the G1 group (upper, arrowheads) than the G5 group (lower, arrowheads) without statistical significance. (D) The number of brown-colored stained endothelial cells on CD31 staining (×200) decreased in the G1 group (upper, arrowheads) than the G5 group (lower, arrowheads).

The MVD of the G1 and G2 group decreased slightly compared to that of the control group (*P* = 0.009 and *P* = 0.012). However, the other groups did not differ significantly compared to the control group (*P* > 0.05) **(Table 4)**. In terms of the apoptotic index, the sorafenib treated group (G2 group) showed lower apoptotic index than the doxorubicin treated group (G4 group) (*P* = 0.039). There was a slightly higher apoptotic index in the G1 group than the G5 group, but there was no statistical significance (*P* > 0.05). The representative case is shown in the **Fig 5**.

**Table 4:** Histologic parameters.

Variable	G1 (N = 10)	G2 (N = 10)	G3 (N = 6)	G4 (N = 7)	G5 (N = 6)	*P*-value*
**Apoptosis**	27.8 ± 8.5	20.9 ± 11.3	31.2 ± 11.8	35.0 ± 7.7	20.8 ± 3.5	**0.019**
*P*-value**	1.000/1.000/1.000/1.000^†^	0.387/**0.039**/1.000^††^	1.000/0.599^†††^	0.091^††††^	-	
**MVD**	10.3 ± 5.8	10.6 ± 4.5	19.2 ± 5.9	16.2 ± 7.5	21.0 ± 4.7	**0.001**
*P*-value**	1.000/0.050/0.439/**0.009**^†^	0.063/0.535/**0.012**^††^	1.000/1.000^†††^	1.000^††††^	-	

Values are mean ± SD. G1, combined sorafenib and doxorubicin-microbubble group; G2, sorafenib-treated group; G3, doxorubicin-microbubble group; G4, doxorubicin treated group; G5, untreated control group; MVD, microvessel density.

*One-way analysis of variance (ANOVA)

**Post-hoc analysis of ANOVA

^**†**^G1 vs. G2 / G1 vs. G3 / G1 vs. G4 / G1 vs. G5

^**††**^G2 vs. G3 / G2 vs. G4 / G2 vs. G5

^**†††**^G3 vs. G4 / G3 vs. G5

^**††††**^G4 vs. G5.

## Discussion

Our results showed that the combination of SOR and DOX-MAC could affect the tumor growth inhibition by the anti-angiogenic and the cytotoxic effect of SOR and DOX-MAC, respectively, in a rat model. We could monitor these changes using CEUS with MB that there was a decrease of nPE and nWiAUC values and an induction of the anti-angiogenetic effect of SOR, which was demonstrated by a decrease of MVD.

Advanced HCC is a devastating disease characterized by a poor prognosis. DOX had a remained issue about the drug resistance and its irreversible toxicity despite one of drug choice for the treatment of advanced HCC [2]. In recent years, SOR, an oral multiple kinase inhibitor targeting the vascular endothelial growth factor (VEGF), was a targeted treatment drug for advanced HCC, but there had been a low treatment response in the majority of patients during the clinical trials [17]. A previous report had reported that the combined treatment with DOX and SOR would be desirable for advanced HCC therapy because there was the possibility of inhibition for the expression of multidrug resistance gene-1 associated with DOX resistance [18,19]. In our study, we observed combined treatment with SOR and DOX-MAC to be more effective than the other therapies at the two-week follow-up, according to the tumor growth analysis. Our study also showed that combination treatment could lead to a decrease in the nPE and nWiAUC values over time than the baseline values.

In fact, SOR has been recommended as the first-line treatment for advanced HCC used to target the tumor vasculature as well as tumor cell viability, which would be a correlation with the inhibiting effect for tumor vessel proliferation [15,20]. Fröhlich et al. [21] had reported that the CEUS perfusion imaging could predict tumor vascular responses after the antiangiogenic therapy before when morphologic changes became apparent change. Their study showed that the perfusion parameters, such as the PE and AUC value, could be used to predict the tumor vascular response after anti-angiogenic treatment. Lassau et al. [22] had demonstrated that the perfusion parameters were significantly associated with disease-free survival in patients with advanced renal cell carcinoma. The perfusion parameters, such as PE, WiAUC, and WoAUC values, could be a useful monitoring tool of the antiangiogenic drug effect [23,24]. However, the reproducibility of the dynamic CEUS parameters would have some limitations and dependent on a variety of settings such as the respiration artifact or tumor depth [25,26]. To overcome these problems, we used the function of in-software automatic motion correction and normalized the TIC value of the tumor using the normal liver parenchyma value of the center depth of the cancer. Therefore, we could demonstrate the nPE and nWiAUC value decrease after combined treatment with SOR and DOX-MAC, although we could not show any changes in the perfusion parameters in the other treatment groups. Our study also showed the inhibition effect of neovascularization with evidence of a decrease of MVD in the combination therapy.

Theragnostic agents are a promising clinical application strategy for simultaneous diagnosis and treatment of HCC. These methods might be an alternative way to treat HCC, considering the side effects of traditional chemotherapy and the poor curative outcome of drug-loaded MBs. Ultrasound-guided drug delivery with MBs has been proposed as a novel approach for chemotherapy drug delivery to HCC because it allows local delivery of chemo-agents into a tumor while minimizing systemic dose and toxicity [27]. Enhanced delivery of therapeutic agents has a mechanism called sonoporation in which a transient opening is formed in the walls of blood vessels by CEUS-triggered oscillation in the region of interest [28]. Several animal studies have shown that CEUS for MB-mediated DOX delivery can be successfully applied in therapeutic applications [27,29–31]. It is also known that drugs combined with MBs and nanoliposome can increase the drug-loading capacity compared to MB alone [32]. However, it might be possible the existence of conjugation with DOX and MB induced changes in the MB surface, leading to the changes of shell elasticity and stiffness and therefore affected the different performances of CEUS imaging [6]. To confirm whether two kinds of contrast agents might have different performance, it can check the enhancement degree of CEUS images of large vessels such as the inferior vena cava during intravenous administration. Interestingly, echogenicity of both DOX-MAC and MAC demonstrated a similar degree of enhancement in the inferior vena cava of all rats. The large size of DOX-MAC is mostly due to the MAC's microbubble size, as shown in Fig 1. Due to this point, the DOX-MAC does not easily extravasate due to the large size of the MBs and is capable of safely delivering DOX to the target site without mid-way losses until the target site is exposed to ultrasound irradiation.

In terms of drug delivery, our data showed a trend of tumor growth suppression and the decrease of the nPE and nWiAUC values compared to those of the control group. However, we could not demonstrate a significant difference in the SOR, DOX-MAC or DOX groups compared to the control group. Tinkov et al. [33] had reported that the DOX-MB compound showed a 12-**fold** increase in DOX uptake by tumor tissue with the application of ultrasound. Our previous studies also showed that DOX-MAC had an advantage regarding tumor treatment in animal studies despite conjugation with a different chemotherapy drug [6,8]. DOX-MAC, as used in our study, had the albumin nanoparticle as a drug carrier to effectively extravasate into the tumor region under the ultrasound exposure and to release doxorubicin in the acidic environment around the tumor [6]. One hypothesis of the failed statistical significance was that circulating DOX-MAC remains in the target region of ultrasound for short periods (seconds), whereas the typical exposure times required for ultrasound-triggered release are much longer (minutes) [32]. The primary mechanism of drug delivery *in vivo* is to passively deliver the circulating agent into the desired tissue. Therefore, our study could not demonstrate that control over drug release timing is a critical aspect of drug delivery and is a necessary part of a rational design approach to optimize chemotherapeutic treatments. It is also possible that drug delivery did not work effectively when tumor blood flow supplied only the peripheral portion of a hepatoma.

Our study did not demonstrate the superiority of DOX-MAC treatment compared to DOX alone or SOR alone, and which is a limitation of this study. However, the SOR + DOX-MAC showed less increase in tumor volume compared to the SOR only treatment at 2-week (16.8 ± 3.8 vs. 19.7 ± 3.2 mm^3^). Also, the DOX-MAC group showed less increase in tumor volume compared to the DOX only treatment (20.5 ± 7.9 vs. 24.5 ± 4.1 mm^3^). These difference in tumor volume might show each method's therapeutic effect. As only numerous comparisons by this statistical test, it cannot be said that the combination of the DOX + SOR method has no therapeutic effect compared to conventional SOR alone or DOX alone treatment. Even though there was a lower MVD of the G1 and G2 group than the control group, the G1 group showed a slightly increased apoptotic index, which might be a possibility of the combined treatment effect with DOX cytotoxicity and SOR anti-angiogenic effect. The G2 group, SOR only treatment, showed a lower apoptotic index than the G4 group, despite lower MVD than the control group, suggesting a lower cytotoxic effect of the SOR. The combination of DOX-MAC and SOR revealed higher apoptosis than SOR alone treatment, which may be associated with the synergistic effects of DOX-MAC and SOR (27.8 ± 8.5 vs. 20.9 ± 11.3), despite the similar MVD between the DOX-MAC and SOR group and SOR alone group (10.3 ± 5.8 vs. 10.6 ± 4.5). These results also could not have statistical power due to multiple comparisons in our study group. The G3 and G4 groups did not show any difference in MVD compared to the control group, which means the less effective anti-angiogenic treatment. Therefore, our study might represent the possibility of the combination treatment effect using DOX-MAC and SOR, rather than the SOR or DOX only treatment.

Our study has some limitations. First, the DOX concentration in our study was designed regarding the tolerance of the rat to our treatment and which was not equivalent to the human dose 20 mg/m^2^. If DOX has reached the therapeutic dose, the side effects of doxorubicin, such as cardiotoxicity, weight loss, alopecia, or neutropenia, may have occurred. However, we could only monitor the weight loss of the rat in our experimental setting. Because there was no weight loss in all experiment groups, we could not convince the adverse effect in the therapeutic dose of DOX or SOR, and explain the adverse event according to the treatment methods. Further studies would be required to determine the optimal DOX concentration that produces effective treatment effects with a negligible toxic effect. Second, in our experimental setting, we did not have the tools for measuring the DOX concentration in the tumor. Even though there was a significant difference between the G4 and the G5 groups of the apoptotic index, further studies regarding DOX quantification, such as using confocal microscopy or liquid chromatography mass spectrometry, should address these issues in order to show the release concentration of DOX-MAC. Despite these limitations, to our knowledge there are no dedicated studies regarding the therapeutic effect of combined SOR and DOX-MAC treatment in a rat orthotopic model of human HCC. Third, it should be mentioned the weak point about the results of perfusion parameters for comparison between groups. We failed the perfusion parameter analysis in the large number of rats (38.1%). The pitfall of CEUS images might be the limited time of image capture because of the large ultrasound imaging file size. All images of the DOX-MAC or MAC agent entering and disappearing from the tumor and liver parenchyma should be saved for appropriate perfusion analysis. However, the 90 seconds might be too short to save all images in which the contrast agent was disappeared in the liver parenchyma. Therefore, we failed the many cases to acquire the appropriate wash-out perfusion parameters. Also, if the tumor volume increased after 2 weeks, it was difficult to obtain consistent perfusion images. In most cases where the image analysis failed, the second-week perfusion image could not be obtained, resulting in no continuous statistical analysis result per subject.

In conclusion, the combined SOR and DOX-MAC treatments could affect the tumor growth inhibition in the rat model of human HCC, and CEUS perfusion parameters can be a useful marker for monitoring the therapeutic response following the treatment.
